# Crystal structure of the Z-ring associated cell division protein ZapC from *Escherichia coli*

**DOI:** 10.1016/j.febslet.2015.11.030

**Published:** 2015-12-21

**Authors:** Cristina Ortiz, Danguole Kureisaite-Ciziene, Florian Schmitz, Stephen H. McLaughlin, Miguel Vicente, Jan Löwe

**Affiliations:** a MRC Laboratory of Molecular Biology, Francis Crick Avenue, Cambridge CB2 0QH, UK; b Centro Nacional de Biotecnología, CSIC C/ Darwin 3, 28049 Madrid, Spain

**Keywords:** Bacterial cell division, FtsZ, Divisome, Z-ring, Tudor domain, Chromo domain

## Abstract

Bacterial cell division involves a contractile ring that organises downstream proteins at the division site and which contains the tubulin homologue FtsZ. ZapC has been discovered as a non-essential regulator of FtsZ. It localises to the septal ring and deletion of *zapC* leads to a mild phenotype, while overexpression inhibits cell division. Interference with cell division is facilitated by an interaction with FtsZ. Here, we present the 2.9 Å crystal structure of ZapC from *Escherichia coli*. ZapC forms a dimer and comprises two domains that belong to the Royal superfamily of which many members bind methylated arginines or lysines. ZapC contains an N-terminal chromo-like domain and a Tudor-like C-terminal domain. We show by ITC that ZapC binds the C-terminal tail of FtsZ.

## Highlights

• First crystal structure of bacterial cell division regulator ZapC solved.
• ZapC is a two-domain protein, with similarities to Tudor and chromo domains.
• ZapC binds the C-terminal tail of FtsZ with moderate affinity.

## 1 Introduction

Most bacteria and archaea divide using a contractile septal ring that is composed of intracellular or inner, cytosolic proteins, including the bacterial tubulin homologue FtsZ, and also middle, transmembrane proteins and outer, periplasmic proteins [1], [2], [3]. Together, the putative protein complexes involved in this process have been termed the divisome [4]. The inner divisome proteins organise into a ring structure, the Z-ring that is used as a platform to recruit the downstream components of the system including the transmembrane and periplasmic proteins [5]. Several divisome components are essential for septation and their inactivity or absence prevents division, causing growth as filamentous cells [6].

In *Escherichia coli*, the inner proto-ring proteins [7], FtsZ, FtsA and ZipA, are essential. Transmembrane or middle divisome proteins facilitate communication between the inside of the cell and the periplasm, where the cell wall is synthesised and in *E. coli* include the essential components FtsB, FtsI, FtsL, FtsK, FtsN, FtsQ and FtsW [8]. Together, inner and middle divisome components interact with outer periplasmic proteins that facilitate or organise the remodelling of the cell wall and outer envelope, for example PBPs (penicillin binding proteins), amidases and autolysins [9]. Remodelling of the cell wall, inner membrane constriction and Z-ring constriction normally occur concurrently and it is not known what the force generators for constriction are and if there is more than one, how they are synchronised.

Apart from essential components, the number of non-essential divisome components being discovered is increasing, including new elements in the inner proto-ring [10]. Several proteins have been discovered that localise to the site of constriction and show weak division-defective phenotypes on their own and stronger phenotypes when combined, possibly indicating a role in making the process more robust or efficient [11]. Such proteins include the Zap proteins (Z-ring Associated Proteins), ZapA [12], ZapB [13], ZapC [14], [15] and ZapD [16]. These proteins do not have much in common and, apart from ZapA and ZapB, do not seem to function together. ZapA, ZapC and ZapD bind to FtsZ directly and affect FtsZ polymerisation and bundling in vitro [12], [14], [15], [16].

ZapC was discovered using Z-ring fluorescence localisation screens [8] and through transposon screens in the absence of a functional Min system [9]. ZapC has no clear homologues of known function, it is 180 residues long in *E. coli* and is found across gammaproteobacterial species [14]. When deleted, ZapC causes a mild division-defective phenotype, but when overexpressed, ZapC causes a severe cell division defect leading to filamentous growth [14], [15]. ZapC interacts with FtsZ and its intracellular localisation is only dependent on FtsZ and none of the other downstream divisome components [14], [15]. Mixing of ZapC with FtsZ leads to increased FtsZ filament bundling when observed by negative-stain electron microscopy and light scattering. A ZapC mutant L22P has been described that abrogates the FtsZ interaction in vitro, as well as the cell division phenotypes upon deletion and overexpression [15]. Recently, ZapC has been shown to interact with the body of FtsZ, not the C-terminal tail, leading to nanomolar affinity between the two proteins as measured by fluorescence-based bulk pelleting assays, pulling labelled ZapC into FtsZ polymers by centrifugation [17]. It has been suggested that ZapC is a monomer based on calibrated size exclusion chromatography [15].

In this work we present the 2.9 Å crystal structure of ZapC from *E. coli*, showing that it is composed of two distinct domains belonging to the chromo and Tudor domain families. We find that ZapC is a weak dimer and also shows moderate affinity for a peptide derived from the C-terminal tail of FtsZ.

## 2 Materials and methods

### 2.1 Cloning, protein expression and purification

The *E. coli* K12 *zapC* gene (GenBank: AAC74032.2, coding for UniProt protein sequence P75862.2) was amplified using PCR from genomic DNA, cloned into the NdeI/BamHI sites of plasmid pHis17 (Bruno Miroux, personal communication; small, pET derived plasmid with T7 promoter) and was expressed as a C-terminal His_6_-fusion in *E. coli* C41(DE3) cells (Lucigen). According to established feed-back inhibition protocols [18], [19], selenomethionine-substituted (SeMet) ZapC cultures were grown to early log phase (OD_600_ = 0.6) at 37 °C in M9 minimal medium, supplemented with 0.4% (w/v) glucose, 2 mM MgSO_4_. 100 mg/L of dl-selenomethionine (Generon), 100 mg/L of lysine, threonine, phenylalanine and 50 mg/L of leucine, isoleucine and valine were added as solids at the same time. Fifteen minutes later, protein expression was induced with 0.5 mM isopropyl-β-d-thiogalactopyranoside (IPTG) and cells were further grown overnight at 20 °C. Cells were harvested by centrifugation at 4000 rpm for 15 min. The pellet was re-suspended in 50 mM CHES, 300 mM NaCl, 15% glycerol (v/v), 2 mM DTT, pH 9.0. Cell lysis was carried out at 25 kPSI using a cell disruptor system (Constant Systems, Daventry, UK) and the lysate was clarified by centrifugation at 30 000 rpm for 30 min at 4 °C. The supernatant was passed over a 5 mL HisTrap HP column (GE Healthcare). The column was equilibrated with 25 mM CHES, 300 mM NaCl, 10% glycerol (v/v), 1 mM DTT, pH 9.0. ZapC was eluted with 100–300 mM imidazole in the same buffer. Peak fractions were concentrated with centrifugal concentrators (Vivaspin, Sartorius) and loaded onto a HiLoad Sephacryl S200 16/60 column (GE Healthcare) equilibrated in 20 mM CHES, 250 mM NaCl, 10% glycerol, 5 mM DTT, pH 9.0. Purified ZapC was concentrated as before to 5 mg/mL for immediate use without freezing.

Untagged *E. coli* full-length ZapC protein was cloned the same way with a stop codon in place of the histine tag and overproduced from 12 L C41(DE3) bacterial culture grown at 37 °C to an optical density OD_600_ of 0.5, at which point expression of ZapC was induced with 1 mM IPTG for 3 h at 37 °C. Cells were centrifuged and pellets were resuspended in buffer A (50 mM Tris–HCl, 50 mM KCl, 10% glycerol, pH 8.0), supplemented with DNAse I (Sigma) and protease inhibitor tablets (Roche, Germany). Cell lysate was passed through a Constant Systems cell disruptor at 25 kPSI and the lysate was centrifuged for 30 min at 40 000 rpm in a Beckman Ti 45 rotor at 4 °C. The supernatant was loaded onto a 5 mL HiTrap Q HP column (GE Healthcare), previously equilibrated with Buffer A. The column was washed and ZapC was eluted in a gradient to 1 M KCl in buffer A. ZapC eluted at around 300 mM KCl. Peak fractions were pooled and concentrated to be loaded onto a HiPrep 16/60 Sephacryl S200 column (GE Healthcare) equilibrated in 50 mM Tris–HCl, 250 mM KCl, 1 mM EDTA, 10% glycerol, pH 7.5. The pooled fractions were concentrated to 3 mg/mL, aliquoted and stored frozen at −80 °C.

### 2.2 Crystallisation

Crystallisation conditions were found using our in house high-throughput crystallisation platform [20], mixing 100 nL SeMet ZapC solution at 5 mg/mL with 100 nL of 1920 different crystallisation reagents in MRC vapour diffusion sitting drop crystallisation plates. For data collection, SeMet-substituted ZapC crystals were grown at 19 °C by using the following final crystallisation solution: 0.58 M ammonium tartrate, 0.01 M sodium acetate, pH 4.6, and this was mixed again 1:1 with protein solution. Crystals were flash frozen for data collection after adding 20% (v/v) glycerol as a cryo-protectant.

### 2.3 Structure determination

Since crystals were thin, long needles we used DLS’s (Diamond Light Source, Harwell, UK) micro focus beamline I24 for data collection. Using un-attenuated beam and 100 ms exposure time, good diffraction to better than 3.0 Å at the selenium edge could be achieved but crystals were damaged rapidly under those conditions. We therefore adopted a data collection strategy by which 16 wedges from 3 independent crystals were collected, translating the beam along the crystals for the wedges that came from the same crystals. In this way, a complete and highly redundant dataset to 2.9 Å with very good anomalous signal was obtained (Table 1). Selenium sites were found with SHELXCD [21] and initial maps were obtained by PHASER in SAD mode [22]. It was immediately recognised that the electron density maps contain a local twofold axis and the axis was localised using PHENIX.FIND_NCS [23]. Adding the NCS symmetry to solvent flattening after PHASER produced a good map that BUCCANEER [24] could build into. BUCCANEER was forced to recognise the local twofold axis by re-arranging chains manually and finally REFMAC [25] and manual model building/correcting in MAIN 2014 [26] were employed to produce a fully refined model of the *E. coli* ZapC dimer. Data collection and refinement statistics are listed in Table 1. The experimental structure factors and refined coordinates have been deposited in the Protein Data Bank (PDB) with accession code 5fo3.

### 2.4 Isothermal titration calorimetry

Isothermal titration calorimetry (ITC) experiments were carried out using a MicroCal iTC200 instrument (Malvern Instruments, Malvern, UK) at 20 °C. Titrations consisted of 19 consecutive 2 μL injections of protein ligand (following a pre-injection of 0.5 μL) into the buffer or protein sample in the cell at 120 s intervals. Un-tagged ZapC was used for ITC. Experiments consisted of injecting 2 mM FtsZ peptide into ZapC (100 μM) in 50 mM Tris–HCl, 150 mM KCl, 1 mM EDTA, 2 mM MgCl_2_, pH 7.4. The resulting integrated heats were corrected for the heat of dilution, then fitted to a one-site binding model and binding constants calculated using the Origin software (OriginLab, MA).

### 2.5 Analytical ultracentrifugation

Proteins samples (full-length, un-tagged) at concentrations of 1–3 mg/mL in 50 mM Tris–HCl, 250 mM KCl, 1 mM EDTA, 10% (v/v) glycerol, pH 7.5) were centrifuged at 50 000 rpm at 20 °C using 12 mm double sector cells in an An60Ti rotor, while monitoring interference in a Beckman Optima XL-I analytical ultracentrifuge. The sedimentation coefficient distribution function, c(s), was analysed using the SEDFIT program, version 13.0 [27] with a frictional ratio (*f*/*f_o_*) of 1.20–1.23. The partial-specific volume (v-bar), solvent density and viscosity were calculated using SEDNTERP [28]. The calculated v-bar was corrected for the effect of the presence of glycerol using the below formula, derived from the data of Gekko and Timasheff [29]. Data were plotted using GUSSI (biophysics.swmed.edu/MBR/software.html).

$$\frac{\Delta \text{vbar}}{\Delta \%\;\text{vol glycerol}}=3.33\times 10^{-4}$$

## 3 Results and discussion

**Fig. 1:**
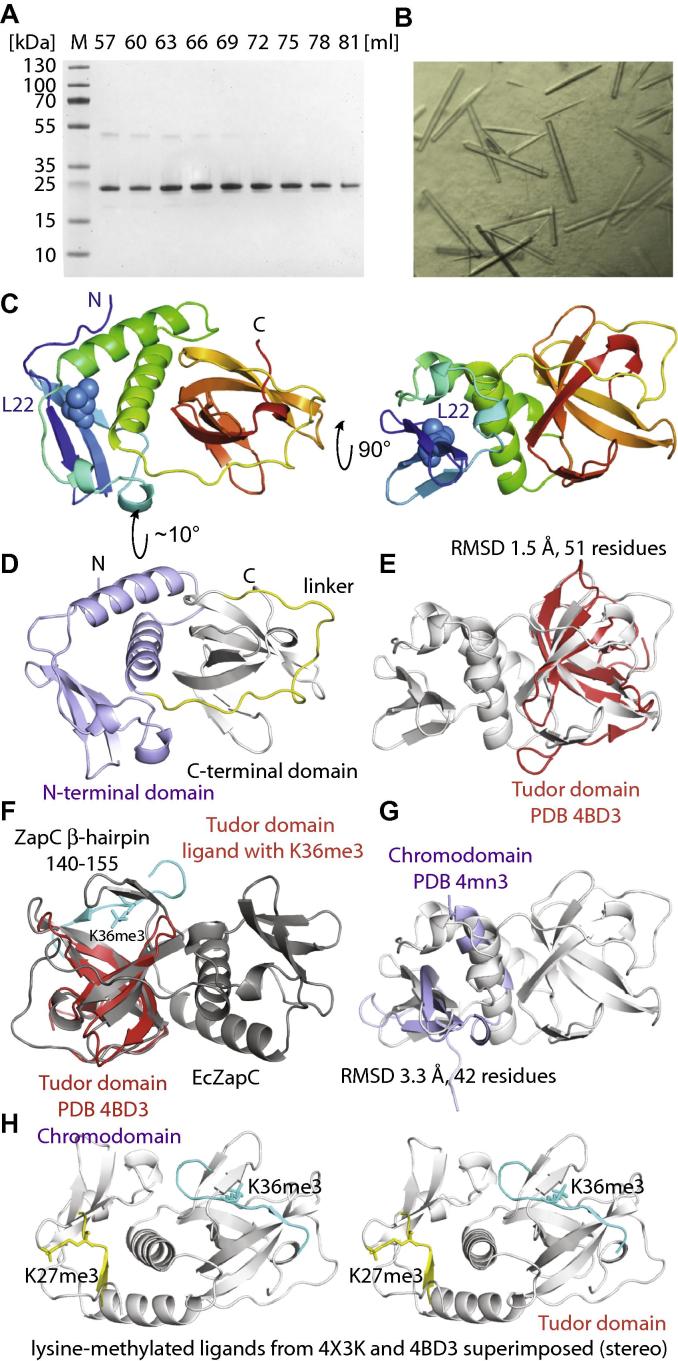
(A) Coomassie-stained SDS–PAGE gel showing fractions after size exclusion chromatography of *E. coli* SeMet ZapC. The first two fractions correspond to the void volume of the column used (see Section 2). (B) Typical crystals of *E. coli* SeMet ZapC after optimisation as used in this study. The needles were typically less than 20 μm in diameter, leading to weak diffraction and strong decay in the X-ray beam during data collection. Several wedges were collected from three crystals while translating along the long axis of the crystals and merged for structure determination. (C) Ribbon plot (PyMOL, Schrödinger) outlining the crystal structure of ZapC at 2.9 Å resolution. The structure is shown in rainbow colours from the N-terminus in blue to the C-terminus in red. Amino acid L22 is indicated by spheres [9]. (D) ZapC contains two fairly separate domains that are connected via a long linker, shown in yellow. The N-terminal domain is shown in light blue, the C-terminal domain in grey. (E) The C-terminal domain of ZapC is related to Tudor domains. Many such related domains come up in database structural similarity searches (DALI). A superposition between PDB 4BD3 (PHD finger protein 19, red, RMSD 1.5 Å over 51 Cα) [24] and ZapC’s C-terminal Tudor domain is shown. (F) Tudor domains are known to preferentially bind to peptides containing methylated arginines and lysines. The superposition in E is repeated here with the methylated (K36me3) histone tail peptide ligand shown in cyan. It is clear that the same binding pocket in ZapC is occluded by a small beta-hairpin comprising residues 141–154. (G) The N-terminal domain of ZapC is distantly related to chromo domains. Again, many such domains come up in database searches (PDBe FOLD). A superposition between PDB 4MN3 (CBX7, chromobox homologue 7, light blue, RMSD 3.3 Å over 42 Cα) [28] and ZapC’s chromo domain is shown. (H) Stereo plot showing both superpositions (from panels E, F; and G) with their lysine-methylated peptide ligands, only (yellow: chromo domain, cyan: Tudor domain). As for the Tudor domain, the canonical peptide pocket in ZapC’s chromo domain is probably occluded by a different orientation of the first strand and a small helical segment between residues 44 and 50.

We cloned and expressed *zapC* from *E. coli* in *E. coli*, adding a histidine tag at the C-terminus for crystallography, only since many purifications had to be performed. After purification (Fig.1A) we routinely produced around 5 mg ZapC protein from 12 L of culture for both the SeMet and native ZapC proteins. ZapC proved to be difficult to handle in most buffers but we found it to be manageable in a buffer at high pH (Section 2). Crystallisation trials yielded needle-shaped crystals (Fig.1B) that diffracted well enough but were difficult to grow bigger. Structure determination from seleno-methionine substituted crystals by SeMet SAD hence utilised a microfocus beamline (I24, Diamond Light Source, Harwell, UK), using three needle-shaped crystals and 16 wedges collected from different spots along the crystals. This yielded a highly redundant dataset suitable for phasing. Phasing, automated and manual model building, and refinement produced an atomic model of very good quality, resolving residues 1–168 of ZapC, with two molecules in the asymmetric unit, at 2.9 Å resolution (Table 1).

The structure of the ZapC monomer (Fig.1C) shows it to be composed of alpha helices and two small beta sheets. The protein contains two separate domains that are linked by a long, extended and irregular linker reaching almost all the way around the molecule (residues 88–109). This is presumably because the folds of the two domains place their C- and N-termini at opposite ends of the molecule (Fig.1D, linker in yellow).

Overproduction of ZapC shows strong inhibition of cell division in *E. coli* and it was previously shown that a mutation, (L22P), abolishes this effect [15]. Using mutant protein carrying the same mutation also had a negative effect in a number of in vitro interaction assays with FtsZ. The position of this mutant in the middle of the second beta strand of the small beta sheet of the N-terminal domain is shown in Fig.1C. Given that L22 was mutated to proline, we think it is very likely that this change disrupts folding in this region of the protein as prolines do not support regular beta sheet formation and the side chain of L22 points into the hydrophobic core of the N-terminal domain. It might also modulate dimer formation as mentioned later.

The crystal structure shows that the C-terminal domain of ZapC is related to Tudor domains. For example, ZapC can be superimposed on subunit Phf19 of the polycomb repressive complex 2 (PRC2, PDB 4BD3) [30] with an RMSD of 1.5 Å over 51 Cα atoms (Fig.1E). In fact, all top DALI [31] structural similarity search hits were Tudor domains, indicating a high significance of this finding. Although the fit is good, ZapC contains an extra beta hairpin (residues 141–154) that is not normally present in Tudor domains [32] (Fig.1F).

The N-terminal domain is distantly related to chromo domains as was found when comparing ZapC to all entries in the Protein Data Bank (PDB) with PDBe Fold (SSM) [33]. For example, the N-terminal domain of ZapC can be superimposed on chromobox homolog 7 (CBX7, PDB 4MN3) [34] with an RMSD of 3.3 Å over 42 Cα atoms (Fig.1G). The fit is not as good as for the C-terminal domain and Tudor domains, and the angles of all secondary structural elements are slightly different between chromo domains and ZapC-N. When compared to canonical chromo domains, an extra helical loop and helix are found in the ZapC N-terminal domain (residues 32–71). The last helix of the ZapC chromo domain (residues 72–87) is longer than in canonical chromo domains and is connected *via* the long linker (Fig.1C) to the N-terminus of ZapC’s Tudor domain.

Tudor domains and chromo domains belong to the so-called Royal superfamily that comprises Tudor, chromo, MBT, PWWP and Agenet domains [32]. Interestingly, these domains often bind methylated lysines and arginines and they are probably best known for their sensing of methylated histone tails in eukaryotes. This means that ZapC contains two distantly related domains that are part of a superfamily of domains known for their ability to sense arginine- and lysine-methylated peptides.

We investigated the structure of the putative canonical peptide binding sites in both the N-terminal chromo domain and C-terminal Tudor domain. In both cases, the binding sites seem to be occluded. In the Tudor domain of ZapC, the extra beta hairpin (residues 141–154) covers the very hydrophobic pocket that in canonical Tudor domains binds the methylated lysine-containing peptide (K36me3, Fig.1F, cyan peptide). In the N-terminal chromo domain of ZapC, the binding site is not quite as occluded but the hydrophobic pocket is not very accessible either (Fig.1H, yellow peptide containing K27me3). In both cases, however, it seems plausible that the domains could eventually undergo conformational changes allowing access to the hydrophobic pockets that are required for methylated arginine and lysine binding.

**Fig. 2:**
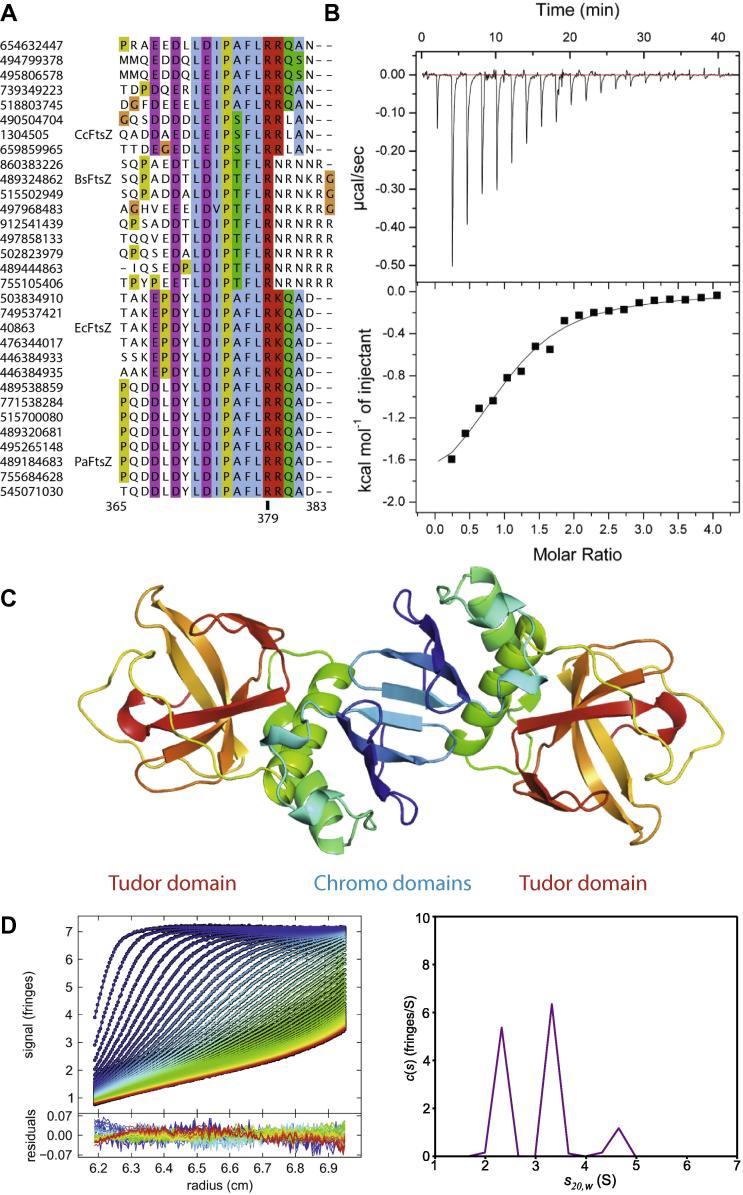
(A) Multiple sequence alignment (Clustal Omega, www.clustal.org) showing conservation within the C-terminal tails of various FtsZ proteins. A maximum of ten sequences were collected from each BLAST search with FtsZ sequences from *E. coli*, *B. subtilis*, *C. crescentus* and *P. aeruginosa* (Ec, Bs, Cc, PaFtsZ) sampled evenly up to the point where percentage of sequence cover and identify dropped below 95% and 40%, respectively. Totally conserved arginine residue 379 is highlighted; numbering corresponds to EcFtsZ. (B) Binding of FtsZ peptide to ZapC. Raw heats measured during injections of FtsZ peptide into ZapC by ITC (top) were integrated and fitted to a single-site binding model (bottom) yielding a stoichiometry of 1.0 with an enthalpy of −2.1 kcal/mol and *K_D_* of 32 ± 6 μM. (C) ZapC dimer as suggested by crystal packing and PISA (PDBe PISA; www.ebi.ac.uk/pdbe/pisa) [30]. The strands of the N-terminal chromo domain come together to form an eight-stranded sheet. (D) Left: analysis of the oligomeric state of ZapC from sedimentation velocity analytical ultracentrifugation. Interference scans (symbols) and best-fit c(s) model at different points in time indicated by colour temperature with residuals to the fit below. Right: c(s) sedimentation coefficient distribution showing peaks for monomer, dimer and higher oligomer.

It appears striking to us that a bacterial cell division protein contains two domains of the Royal superfamily. Could ZapC be involved in binding methylated regions of FtsZ? Previously, it has been suggested that methylation might be required for progression of cell division as determined by the consequences of reducing methylation co-factor availability [35]. The highly conserved C-terminal tail of FtsZ could be a possible site for such post-translation modification as it is spaced away from the folded body of the protein by a long flexible linker and contains a highly conserved arginine, R379 (*E. coli* numbering, Fig.2A). It is very tempting to speculate that binding of the large number of FtsZ-interacting proteins to this tail might be regulated by post-translational modification as otherwise it is difficult to explain how so many different proteins may work in concert while binding to exactly the same FtsZ motif. However this hypothesis has two distinct problems: (i) we are not aware of any methylation reported for FtsZ from any bacterium; and (ii) it has recently been reported that ZapC binds to the body of FtsZ, not the C-terminal peptide [17]. However, this study used mostly pelleting conditions and fluorescence changes as readout, both of which are indirect and prone to problems because the assays involve sedimentation. To investigate this conflicting evidence, we probed the direct interaction of a peptide derived from the C-terminal tail of FtsZ with ZapC by ITC (isothermal titration calorimetry) and obtained a *K_D_* of 32 μM, as expected for binding of a short, flexible peptide (Fig.2A and B). This does not disprove the existence of other ZapC binding surfaces on the body of FtsZ that could lead to the reported nanomolar affinities [17]. Clearly, these matters require further investigation in the future.

The structure of the ZapC crystals described here contains two monomers per asymmetric unit. Although there are two other contacts in the lattice, the one shown in Fig.2C clearly has the largest interface, buries a number of hydrophobic side chains and completes the otherwise open beta sheet of the N-terminal chromo domain forming an eight-stranded beta sheet across the two monomers. It is therefore expected that the previously reported mutation L22P would have an effect on dimerisation, as it was reported to increase the amount of multimers found after size exclusion chromatography. This particular dimer was also selected computationally by PISA (PDBe PISA, http://www.ebi.ac.uk/pdbe/pisa) [36] as the most likely assembly. The dimer interface buries a contact area of approximately 1500 Å^2^ (out of 8900 Å^2^ of each monomer). In order to clarify the oligomeric state of the protein in solution, we performed analytical ultracentrifugation (Fig.2D), finding that ZapC runs as three species. The two major species corresponded very well with theoretical values for ZapC monomers and dimers. There is a minor species of higher S value but this varies between different concentrations corresponding to masses between a trimer and tetramer. This may be due to ZapC aggregation, which we observed during analytical ultracentrifugation in the absence of glycerol (not shown). The concentrations of monomeric and dimeric species can be calculated by integrating the baseline-separated distributions, giving an estimate for the dissociation constant of approximately 30 μM for the monomer–dimer equilibrium. If one FtsZ tail binds one ZapC monomer, as indicated here by ITC (Fig.2B), it is expected that a dimer of ZapC would have the ability to crosslink and bundle FtsZ filaments, as has been reported [14], [15]. Beyond this, we can only speculate what the role of dimerisation is in the context of ZapC’s interaction with FtsZ and in the wider context of bacterial cell division but it is intriguing that a number of FtsZ-interacting proteins are dimers, including ZipA [37], [38], SulA [39], MinC [40], ZapA [41], SlmA [42] and EzrA [43] and most of these also bind to the C-terminal tail of FtsZ.

## Figures and Tables

**Table 1:** Crystallographic data.

Protein	*E. coli* ZapC
UniProt ID	P75862.2

*Data collection*	
Beamline	Diamond I24
Wavelength (Å)	0.97858

*Method crystal*	SeMet SAD
Space group	P4_3_2_1_2
Cell (Å)	87.4, 87.4, 118.3

*Scaling*	
Resolution (Å)	2.9
Completeness (%)^a^	100.0 (100.0)
Multiplicity^a^	41.8 (40.7) 16 wedges from 3 crystals
(*I*)/*σ*(*I*)^a^	17.0 (3.9)
*R*_merge_^a^	0.302 (1.758)
*R*_pim_^a^	0.064 (0.378)
Wilson B-factor (Å^2^)	80.2

*Refinement*	
*R*/*R*_free_^b^	0.213 (0.247)
Model	2 monomers: A: 1–168; B: 1–168, 0 H_2_O
Bond length RMSD (Å)	0.021
Bond angle RMSD (°)	2.555
Average B-factor of all atoms (Å^2^)	51.0
Favoured (%)^c^	99.3
Disallowed (%)^c^	0.0
Molprobity percentile	82nd

PDB ID	5fo3

a Values in parentheses refer to the highest recorded resolution shell.

b 5% of reflections were randomly selected before refinement.

c Percentage of residues in the Ramachandran plot (PROCHECK).
